# Online Discussion and the Moral Pathway to Identity Politicization and Collective Action

**DOI:** 10.5964/ejop.v14i1.1507

**Published:** 2018-03-12

**Authors:** Augusta Isabella Alberici, Patrizia Milesi

**Affiliations:** a Catholic University of the Sacred Heart, Milan, Italy; Department of Psychology, Webster University Geneva, Geneva, Switzerland; London School of Economics, London, United Kingdom

**Keywords:** collective action, computer-mediated communication, moral obligation, online deliberation, online discussion, politicized identity

## Abstract

Research on the mobilizing potential of the Internet has produced some controversy between optimistic vs. skeptical perspectives. Although some attention has been paid to the effects of online discussions on collective participation, very little is known about how people’s experience of online interactions affects the key psychosocial predictors of collective action. The present research investigated whether use of the Internet as a channel for deliberation influenced the moral pathway to collective mobilization by shaping users’ politicized identity, thereby indirectly influencing collective action. Results showed that when people perceived online discussions as a constructive communication context, their politicized identity was imbued with the meaning of responding to a moral obligation, and willingness to participate in collective action was sustained. However, when participants perceived that online discussions were not constructive, their identification with the movement did not refer to moral obligation, and intention to participate in collective action was not sustained. Our discussion focuses on the need to deepen investigation of how people experience the particularities of interacting online, and on how this can affect psychosocial processes leading to collective action.

With the impressive rise of new communication technologies, the mobilizing potential of the Internet has been explored. New communication tools such as Facebook, Twitter, and Instagram have deeply changed the way in which people interact and discuss public issues. A number of studies have investigated the effects of computer-mediated communication (CMC) on political engagement and on willingness to take part in collective actions (e.g. Brunsting & Postmes, 2002; Castells, 1996; Rheingold, 1995), and some controversy has emerged (e.g. Farrell, 2012; Margolis & Resnick, 2000; Sunstein, 2002). According to many, online interactions have the ability to increase offline and conventional forms of political participation (Cho et al., 2009; Mazzoni & Alberici, 2016; Mossberger, Tolbert, & MacNeal, 2008; Rojas & Puig-i-Abril, 2009; Shah, Cho, Eveland, & Kwak, 2005; Xenos & Moy, 2007). The Internet would especially have the potential to support collective action of emerging political groups, as well as peripheral movements previously incapable of political action. The Arab spring protests would be one example (e.g. Kerkhof, Krouwel, Van Stekelenburg, & Klandermans, 2013; Tufekci & Wilson, 2012). It was proposed that, during this activity, social media acted as a catalyst helping activists consensualize about mass opposition to the existing oppressive regimes (McGarty, Thomas, Lala, Smith, & Bliuc, 2014). On the other hand, some scholars are more skeptical and argue that the Internet erodes political engagement, political efficacy and trust (for an extended discussion see Ansolabehere and Iyengar, 1995; Nie, 2001). Some suggest it encourages self-segregation into homogenous and extremist groups (e.g. Douglas, 2007; Wojcieszak, 2011), and fosters pursuit of personal rather than collective benefits (Bennett & Segerberg, 2012; Morozov, 2009). In this manner, it is argued, it stimulates less valuable forms of participation, such as ‘clickactivism’ or ‘slacktivism’ (Gladwell, 2010; Vitak et al., 2011).

Within this line of research, some attention has been placed on the characteristics and implications of online political discussions (Valenzuela, Kim, & Gil de Zuñiga, 2011). Conversations via the Web bear some particularities, such as being mostly text-based, asynchronous, and poor in nonverbal cues. As we will discuss later, research on deliberation practices (i.e. the exchange of rational–critical arguments) has produced some controversy regarding the positive vs. negative relation between online interactions and political learning and engagement (see Min, 2007). Recently, some studies found that the way in which people experience online discussions transforms the effect of the key psychosocial predictors of collective action (Alberici & Milesi, 2013; Kende, van Zomeren, Ujhelyi, & Lantos, 2016). The amount and perceived quality of online discussions seem especially to influence the way in which people develop a sense of politicized identity. This is consistent with the idea that through their discourses people construct and reconstruct their social identities dynamically (Hopkins & Kahani-Hopkins, 2009; Postmes, Haslam, & Swaab, 2005; Reicher, Cassidy, Wolpert, Hopkins, & Levine, 2006). However, very little is known on how people’s perceptions of the specific characteristics of online discussions influence the way in which politicized identities develop, indirectly stimulating (or deterring) willingness to participate in collective actions.

Therefore, the purpose of the current paper is to deepen investigation of the implications of online discussions for collective action, focusing on the role played by a range of specific characteristics of interacting online. Special attention was placed on how online discussions lend moral meaning to activists' sense of politicized identity. Indeed, when activists interact online, they exchange opinions about how the world should change. Otherwise put, they feel a conflict between ‘the way the world is’ and ‘the way the world should be’ (Smith, Gavin, & Sharp, 2015). By consensualizing on this discrepancy, people’s individual sense of moral obligation to act may become a socially validated norm that can contribute to foster politicized identity.

In the following introductory sections, we first focus on the psychosocial predictors of collective action. We then analyze how the unique features of online discussions can impact the development of a sense of politicized identity and, indirectly, the intention to act collectively.

## The Psychosocial Predictors of Collective Action

Collective action can be defined as acts that individuals or psychological group members undertake in a political context to achieve personal or group goals, such as defending their moral principles or improving conditions of an entire disadvantaged group (Thomas, McGarty, Reese, Berndsen, & Bliuc, 2016; van Zomeren, 2016). Psychosocial literature has highlighted various motivations that drive people to join collective action. Among them, a special role is ascribed to politicized group identity, as it plays a pivotal role in connecting other psychosocial motivations to action.

Politicized group identity is based upon group members’ awareness that they share grievances and that their unsatisfying condition can be attributed to an opponent. As compared with non-politicized group identity, politicized group identity has a stronger normative content. Injunctive norms about ‘the way the world should be’, ‘how it should change’, and ‘what we should do to make it change’ lie at the core of its contents. As suggested above, these norms are socially validated and consensualized through intragroup discussion (Postmes, Haslam, & Swaab, 2005; Postmes, Spears, Lee, & Novak, 2005; Smith et al., 2015). As a result of the latter, group members converge on what defines them as a group: their collective interests and goals, the necessity to act collectively, and the specific actions they should undertake as a group. Indeed, group members develop a fully politicized group identity when they become aware they need to bring their fight into the political arena and make other parties (e.g., public opinion) take sides (Simon & Klandermans, 2001). Politicized group identity plays a key role in predicting collective action: the politicization process implies an encapsulation of other motivations to action and, feeding back, it further facilitates them (Thomas, Mavor, & McGarty, 2012; Turner-Zwinkels, Van Zomeren, & Postmes, 2015; van Zomeren, Postmes, & Spears, 2008).

Those motivations include both group-based variables and individual variables. With respect to group-based variables, attention has focused on group efficacy and group-based anger. Group efficacy includes subjective beliefs that members are capable of joining efforts and achieving collective goals, so as to change the situation in the desired direction (van Zomeren et al., 2008; van Zomeren, Spears, Fischer, & Leach, 2004). Group-based anger stems from the appraisal that the status quo is unjust and that actions need to be taken against those responsible for the existing injustice (Leach, Iyer, & Pedersen, 2006; van Zomeren et al., 2004).

In terms of individual variables, recent psychosocial literature has focused on individual differences, such as sociopolitical beliefs or values, life experiences, and personal moral motivations (Duncan, 2012; Milesi & Alberici, 2018; Thomas et al., 2016; van Zomeren, 2013). Personality and life experiences are considered as distal antecedents of politicized group identity; on the contrary, moral motivations have been shown to be directly associated with it. Actually, the very process of politicization relies heavily on the moralization of the collective cause (Milesi & Alberici, 2018; Zaal et al., 2017). Moral motivations include moral convictions and a moral obligation to act. Moral convictions are metacognitions that one’s own individual attitude towards the collective cause is rooted in one’s own moral principles (Skitka & Morgan, 2014). Moral obligation to act corresponds with the feeling that action is what should be done, independent of personal interest, others’ approval, or likelihood of success, because it is one’s personal conscience that demands it (Vilas & Sabucedo, 2012). While both are associated with politicized group identity and, through it, they contribute to predict collective action (Alberici & Milesi, 2016; van Zomeren, Postmes, & Spears, 2012), moral obligation seems to be more closely involved in the politicization of group identity. This is probably because it also interacts with intragroup communication, and implies a stronger commitment to act in a public context (Alberici & Milesi, 2016; Milesi & Alberici, 2018; Vilas & Sabucedo, 2012). We start, then, from the idea that online discussions are a communication context where people, in specific ways, can ascribe a moral meaning to their sense of politicized identity.

## Online Discussion and Collective Action

Discussing politics via the Internet has become a primary feature of contemporary political communication. A growing number of people use social media, online networks and blogs not only to stay informed, but also to exchange opinions, develop common understandings and, in some cases, learn how to mobilize for a collective cause. This is especially true for emerging social movement activists (Brunsting & Postmes, 2002).

As suggested above, according to many (e.g., Brunsting & Postmes, 2002; Shah et al., 2005), online interactions bring the potential to increase civic engagement and collective participation. In this regard, some recent studies found that online interactions may affect people's beliefs and emotions that, in turn, bring them to collective action. Moreover, it has been shown that discussions among group members can contribute to develop a sense of politicized identity, indirectly sustaining collective action (Alberici & Milesi, 2013, 2016). These studies highlighted the importance of taking into account not only how often people discuss online, but also how they perceive the *quality* of online interactions. Some preliminary results showed that those who perceive discussions as being of high quality charge their sense of politicized identity with the idea that collective action responds to an individual moral duty (Alberici & Milesi, 2016). The present research is aimed at deepening such preliminary results by: a) focusing on a larger range of specific characteristics and implications of discussing online, and b) analyzing how activists' perceptions of these characteristics affect the moral meaning of politicized identity. Indeed, the exchange of personal ideas about social reality can convert subjective individual perceptions into socially validated injunctive norms, upon which politicized identity can develop (McGarty et al., 2014; Smith et al., 2015). This, in turn, could indirectly affect the intention (or not) to mobilize collectively.

Compared to face-to-face discussions, online discussions present some specific features: they are mostly text-based, asynchronous, poor in visual and social cues, and often occur under conditions of anonymity (Valenzuela et al., 2012). Some have suggested that the text-based and asynchronous character of online interactions may stimulate more reflexive, rational, and argumentative conversations (Coleman & Gotze, 2001; Stromer-Galley & Wichowski, 2010). Some studies conducted on deliberative interactions showed that deliberation facilitates political participation (Katz, 1992; Warren, 1992). Deliberation corresponds to the process whereby a group of individuals think over a common or public problem and exchange rational and critical arguments about it with the aim of finding a solution that is likely to be agreed upon by those who are involved (Habermas, 1989). To be deliberative, discussions require a number of characteristics, such as rationality and reflexivity. For example, online deliberation is sustained when interactions rely on well-founded arguments (Halpern & Gibbs, 2013). Deliberation also is facilitated when individuals have the chance to learn new perspectives from others. In this regard, as online discussions often rely on networks characterized by weak ties, participants are likely to be provided with non-redundant and diverse information that may stimulate political learning (Valenzuela et al., 2012). Min (2007) compared the effects of face-to-face and online deliberation in experimental settings, and found that the latter was almost as effective as face-to-face deliberation in increasing participants’ political engagement.

There also is evidence, however, that some features of online interactions may present an obstacle to deliberation. Text-based and asynchronous communication has been defined as a ‘poor’ form of communication, where the lack of non-verbal cues can easily produce misinterpretations and misunderstandings (Daft & Lengel, 1986; Walther, 1992). According to this view, text-based online interactions would not be efficient for empathizing with others or for exchanging emotions (Cheshin, Rafaeli, & Bos, 2011). This drawback also is associated with the degree of conversational coherence of online discussions, that is, the extent to which messages relate to prior comments or the initial post (Halpern & Gibbs, 2013).

Thus, on the one hand, some specific features of interacting online may lead to constructive and reflexive discussions where participants exchange and learn well-founded arguments; on the other, however, online discussions may be perceived as being non-constructive. This is due to a low conversational coherence which can foster a lack of understanding among users. Starting from these studies, we aimed at investigating how the way in which activists of a social movement experience online discussions affects: a) their sense of politicized identity and, particularly, b) the perception that collective action responds to an individual moral duty. Our core expectation was that when activists perceive online discussions as being reflexive and argumentative, the sense of moral obligation would be strongly associated with participants’ politicized identity. This, in turn, would indirectly stimulate collective action. From a different standpoint, when activists participate in online discussions where conversational coherence is low and users frequently misunderstand each other, the sense of moral obligation might not be associated with activists' politicized identity. As a consequence, intention to participate in collective action would not be sustained.

## Method

### Research Context

Activists of the Italian Water Movement (Forum Italiano dei Movimenti per l’Acqua) participated in the present research. This movement was started to fight against the liberalization of public water services in Italy. This is a trend that began during the Nineties and ultimately was ratified by a law in 2009. To protest against privatization of water services, citizens’ local committees developed across the country and finally joined together to form the Italian Water Movement. Both the local committees and national Italian Water Movement organized their activities and coordinate their initiatives through the Internet: they developed their own websites and blogs and established a network through Facebook, Twitter, and YouTube accounts. The movement was further successful in promoting offline activities, such as national demonstrations in 2010 and, as well, a 2011 national referendum that demanded complete abrogation of the liberalization law. Voters in this referendum supported rescinding the privatization law. However, no government to date has changed it. The movement has carried on its fight, mainly at the local level. Supporters continue to organize campaigns and public activities and to forward proposals for laws against the liberalization of water management.

### Participants

One hundred and forty-three activists of the Italian Water Movement (90 men, *M*_age_ = 49.2 years, *SD* = 12.5 years) were approached during national meetings and rallies between January and May 2013, and were asked to complete a short questionnaire. All defined themselves as supporters of the Movement.

### Measures

#### Politicized Identity

Politicized identity was measured through four items (e.g. ‘I feel I have much in common with other activists of the Water Movement’) (Cronbach’s α = .90). Responses to this and the following measures were submitted on 7-point scales (1 = *do not agree* to 7 = *totally agree*).

#### Collective Efficacy

Participants were asked to rate their agreement with the following items: ‘I think the Water Movement is able to get public opinion’s attention on issues related to water as a common concern’, ‘United, the Water Movement is able to change the situation in our country’, ‘I think the Water Movement will be kept united in the face of obstacles or difficulties’ (Cronbach’s α = .63).

#### Anger

Anger was measured through the following three items: ‘I get angry when I think that’: a) ‘since July 2011 until now, no provider in the whole country has lowered the water fees’; b) ‘the outcome of the June 2011 referendum has been largely ignored by water providers’; and c) ‘the European law allows privatisation of water management’ (Cronbach’s α = .81).

#### Moral Obligation

Participants rated their agreement with two items: ‘I think that every citizen has the responsibility to fight against privatization of water services’, ‘I feel an inner obligation to defend access to water as a common right’ (*r* = .71, *p* < .001).

#### Frequency of Online Political Discussion

Through seven items, participants rated how often during the last year they had joined online political discussions (e.g. ‘… joining political discussion through Facebook or Twitter’) (1 = *never*; 7 = *very often*) (Cronbach’s α = .90). Only respondents who reported they had participated in online political discussions were invited to answer the following questions about their perception of some specific features of same (*n* = 111).

#### Perceived Characteristics of Online Discussion

Participants were asked to report how often they participated in online discussions where:

- participants exchanged well-founded arguments (‘In your opinion, how often do you participate in online discussions where well-founded arguments are exchanged?’; 1 = *very rarely*; 7 = *very frequently*);
- interactions contained misunderstandings among participants (‘In your opinion, how often do you participate in online discussions where messages contain misunderstandings?’; 1 = *very rarely*; 7 = *very frequently*);
- participants were provided with new information (‘In your opinion, how often do you participate in online discussions where new information emerges?’; 1 = *very rarely*; 7 = *very frequently*);
- interactions were characterised by a low conversational coherence (‘In your opinion, how often do you participate in online discussions where messages are not related to previous comments or the initial post?’; 1 = *very rarely*; 7 = *very frequently*);

#### Collective Action Intention

Collective action intention was measured through three items (‘How often during the upcoming year will you sign online petitions?’, ‘… join offline rallies?’, ‘… contribute to organising public events or marches?’; 1 = *never*; 7 = *very often*) (Cronbach’s α = .81).

## Results

To test whether the four characteristics of online discussions were empirically distinct, we carried out principal axis factoring analyses with oblique rotation. Actually, the factor analysis extracted two factors that predicted 79.55% of the variance. Perceived soundness of argumentation and the perception that online discussions provided new information loaded on the first factor (47.48% of variance explained; both factor loadings were .92), while perceived presence of misunderstandings and perceived conversational coherence loaded on the second factor (32.06% of variance explained; factor loadings ranged from .81 to .86). Thus, while the first factor referred to the perception that online discussions were reflexive and relied on well-founded argumentation, the second factor referred to the perception that online discussions contained misunderstandings and a lack of conversational coherence. Based on this, we computed two separated indexes of these two different dimensions: a) reflexivity and b) lack of conversational coherence. These were then employed in the following analyses.

Table 1 provides means, standard deviations and correlations among all the investigated variables. Perceived reflexivity of online discussions was significantly and positively correlated both with politicized identity and collective action intention. Lack of conversational coherence of online discussion did not show significant correlations with either politicized identity or collective action intention. Moreover, the two perceived dimensions of online discussions did not correlate with each other, indicating that they referred to two conceptually different perceptions of the characteristics of online discussions.

**Table 1:** Descriptive Statistics and Correlations

Variable	*M*	*SD*	1	2	3	4	5	6	7	8
1. Collective efficacy	6.18	.71	-							
2. Anger	6.02	1.09	.29**	-						
3. Moral obligation	6.64	.75	.48***	.07	-					
4. Politicized identity	6.51	.67	.50***	.42***	.35***	-				
5. Frequency of online discussion	4.91	1.54	.30***	-.03	.11	.16*	-			
6. Online reflexivity	4.66	1.18	.36***	.12	.24**	.30**	.53***	-		
7. Lack of online conversational coherence	5.05	1.46	-.10	-.08	-.01	.13	.27**	.13	-	
8. Collective action intention	5.68	1.01	.36***	.30***	.09	.52***	.50***	.40***	.13	-

*p < .05. **p < .01. ***p < .001.

To test the predicted moderating effects of the activists' perceptions of online discussion, we performed separate hierarchical regressions with politicized identity as the criterion. Moral obligation and the other group-based motivations (i.e., anger and collective efficacy) were entered in Step 1, followed by the frequency of online discussion and the perceived characteristics of online discussion in Step 2, and by each interaction term (moral obligation☓perceived reflexivity/lack of conversational coherence of online discussion) in Step 3. All variables were centred before entering the regressions. As shown in Table 2, Step 1 revealed significant main effects for moral obligation, as well as for collective efficacy and anger on politicized identity. Frequency of online discussion and the perceived characteristics of online discussion did not show main effects in Step 2. Step 3 revealed significant effects for both the interaction terms. As predicted, moral obligation interacted positively with perceived reflexivity of online discussion (*B* = .11, *SE* = .04; *p* < .01), and negatively with lack of conversational coherence (*B* = -.15, *SE* = .06; *p* < .01).

**Table 2:** Hierarchical Regressions of Politicized Identity on Moral Obligation, Group-Based Motivations, Frequency of Online Discussion, Perceived Features of Online Discussion, and Interactions.

Step and predictor variable	Step 1^a^	Step 2^b^		Step 3^c^	
	B (SE)	B (SE)	B (SE)	B (SE)	B (SE)
Step 1					
Collective efficacy	.31 (.09)***	.29 (.09)**	.29 (.09)**	.26 (.09)**	.25 (.09)**
Anger	.17 (.04)**	.16 (.05)**	.16 (.05)**	.14 (.05)*	.15 (.05)**
Moral obligation	.16 (.08)*	.17 (.08)*	.17 (.08)**	.27 (.09)**	.22 (.08)**
Step 2					
Frequency of online discussion		.04 (.05)	.06 (.05)	.07 (.05)	.04 (.05)
Online reflexivity		.06 (.05)		.05 (.05)	
Lack of online conversational coherence			.02 (.03)		.05 (.04)
Step 3					
Moral obligation*Online reflexivity				.11 (.04)**	
Moral obligation*Lack of online conversational coherence					-.15 (.06)*
Δ*R*^2^	.31***	.04*	.03	.03*	.05*
Total *R*^2^		.35	.34	.38	.39

^a^Moral obligation and group-based motivations. ^b^Step 1 + Online political discussion, perceived features of online discussion. ^c^Step 2 + each of the two-way interactions between moral obligation and features of online discussion.

*p < .05. **p < .01. ***p < .001.

Simple slope analysis of moral obligation☓reflexivity of online discussion interaction revealed that the influence of moral obligation on politicized identity was significant for respondents who reported participating in online discussions featured by high reflexivity (*B* = .40, *SE* = .1; *p* < .01). It was not significant for participants who reported engaging in discussions featured by low reflexivity (*B* = .13, *SE* = .08; *p* = .11). Consistently, simple slope analysis of the moral obligation☓lack of conversational coherence interaction showed that the influence of moral obligation on politicized identity was not significant for those who reported participating in online discussions featured by low conversational coherence (*B* = .03, *SE* = .09; *p* = .74). It was significant for participants who reported engaging in online discussions featured by high conversational coherence (*B =* .42, *SE* = .13; *p* < .05).

**Figure 1:**
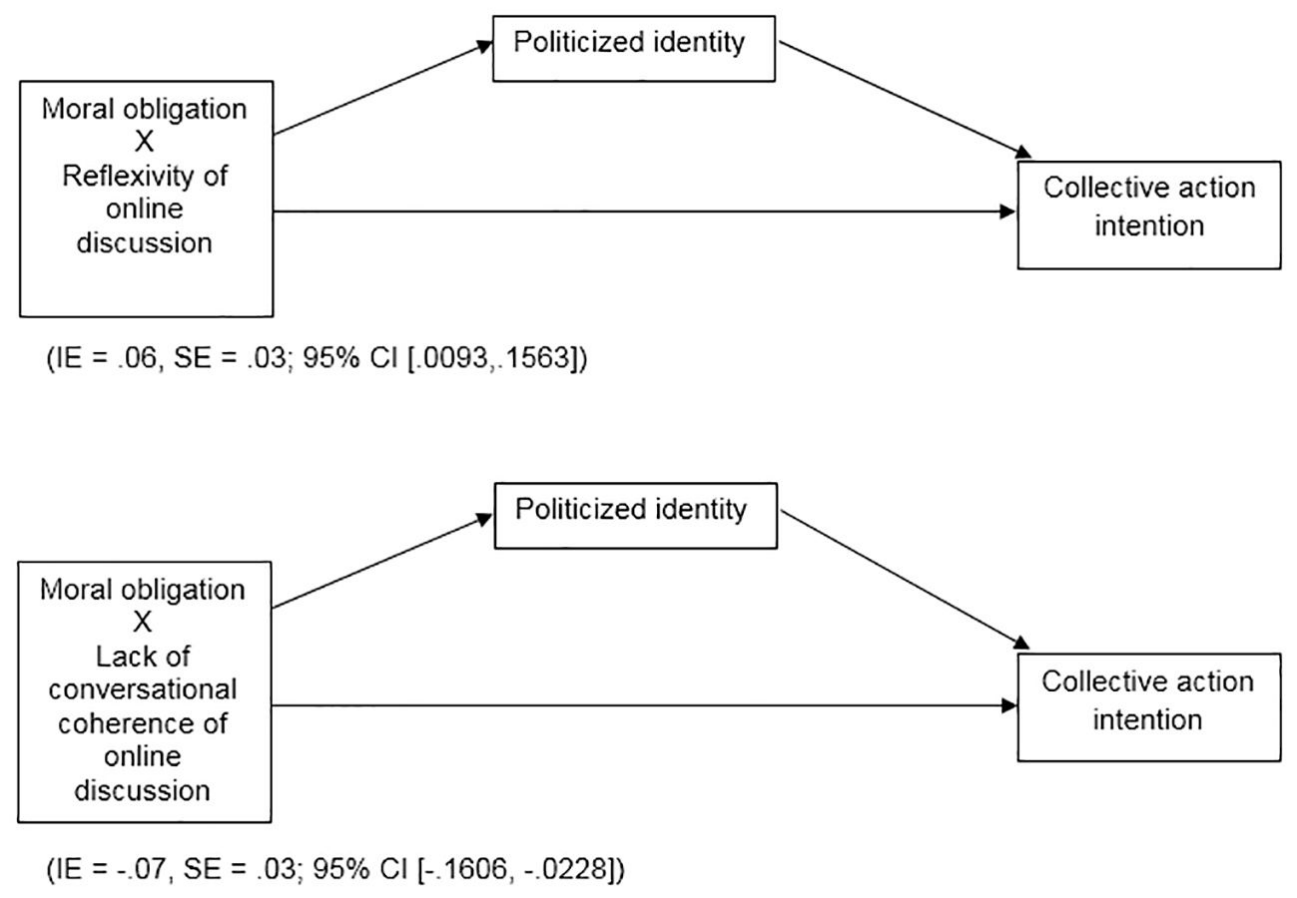
Mediated moderations testing the indirect effects of the emerged interactions (Moral obligation X Reflexivity of online discussion; Moral obligation X Lack of conversational coherence of online discussion) on Collective action intention through the mediation of Politicized identity.

Last, we investigated whether these interactions had an indirect effect on collective action intention through politicized identity. We tested mediated moderation effects for each of the independent variables investigated according to the specifications set out by Hayes’s PROCESS macro for SPSS, using model 8 (Hayes, 2013) (see Figure 1). In each analysis, we controlled frequency of online discussions, collective efficacy, and anger.

First, testing the model for mediated moderation showed that politicized identity mediated the effect of the interaction between reflexivity of online discussions and moral obligation on collective action intention (IE = .06, *SE* = .03; 95% CI [.0093,.1563]). Thus, as expected, the (indirect) effect of moral obligation on intention to mobilize collectively was enhanced when activists participated in reflexive and argumentative online discussions. However, when participants perceived online discussions as being scarcely argumentative, politicized identity did not mediate the effect of moral obligation on collective action. Second, results showed that politicized identity mediated the effect of the interaction between lack of conversational coherence of online discussions and moral obligation on collective action intention (IE = -.07, *SE* = .03; 95% CI [-.1606, -.0228]). Consistent with the first mediated moderation, moral obligation maintained its (indirect) effect on willingness to collective action when activists perceived online discussion was characterised by high conversational coherence. However, when online discussions were perceived as having low conversational coherence, politicized identity did not mediate the effect of moral obligation on intention to collective action. Thus, collective action was not sustained through the investigated path when activists perceived they were participating in non-constructive online discussions.

## Discussion

On the whole, results of our study support the claim that people’s experience of online political discussions can shape the moral pathway to collective action. Findings showed that when activists perceived that the online discussions they joined were a constructive and reflexive communication context, where they exchanged well-founded arguments and learned new information, their identification with the movement was imbued with the sense of responding to a moral duty. In contrast, when participants perceived that online discussions were not a constructive communication context, where there was low conversational coherence and people misunderstood each other, their identification with the movement did not refer to a moral duty. Thus, depending on users’ experience of online discussion, the sense of moral obligation may or may not contribute to shaping the meaning of their politicized identity. Findings also showed that, through this process, perceptions of online discussion can indirectly affect willingness to participate in collective action. Indeed, when activists perceived online discussions as being reflexive, the moral pathway to collective action (through the mediation of politicized identity) was sustained. However, when online discussions were perceived as being poor in conversational coherence, then willingness to take part in collective actions was not stimulated by this path.

Thus, our findings enlarge research on the relation between computer-mediated communication and political participation, and they suggest some possible directions for future research. First, results suggest it is important to investigate not only how interacting online per se affects collective action, but also people’s experience of online discussions. This latter factor, with its specific features, affects the psychosocial processes underlying the development of politicized identity that, in turn, gives rise to action. Our study confirmed that new technologies are not neutral for what concerns effects on collective action (see Spears & Postmes, 2015), in that group identities are influenced and transformed over time during online interactions (Alberici & Milesi, 2016; McGarty et al., 2014). Moreover, by reaching consensus online about the desired social reality, people define their group identities as aimed toward obtaining social change (Thomas, McGarty, & Louis, 2014).

The current study deepens the investigation of how online interactions moderate the moral path to collective action (Mazzoni & Cicognani, 2013; Vilas & Sabucedo, 2012). It seems that when activists interact online, and such interactions support deliberative practices, individual moral judgement about social reality is strengthened. This may contribute to politicizing group identity by reinforcing the idea that there is a moral requirement for struggle in the public arena. The exchange of personal opinions about the desired social reality is likely to convert individual views of moral responsibility in a shared and socially validated norm, which forms the basis for developing a politicized identity. It is also possible that, when people participate in reflexive and well-argued discussions, they are likely to engage in post hoc moral reasoning; that is, they put forward arguments to support an a priori moral judgement (Haidt, 2001). This process would not be sustained, however, when online discussions are perceived to be non-constructive in terms of their conversational coherence.

A further point concerns the importance of more deeply investigating how people experience the specific features of interacting online. As suggested above, online discussions have some unique features, not experienced in face-to-face conversations. Depending on *how* people experience these aspects, online interactions may sustain collective participation on the one hand, but on the other, weaken the psychosocial processes underlying willingness to mobilize. This result could enrich the ongoing debate regarding the mobilizing potential of the Internet. It suggests attention should be focused not only on effects of the amount of online interactions, but also on the ways in which people use and experience new technologies (Kende et al., 2016). In this regard, people may show individual differences in the way they perceive the same online discussions. For example, our results showed that online discussions were perceived to be more or less constructive. Individual interest and knowledge of politics could be relevant here: more interested and expert participants could rely on different standards when they evaluate argumentative quality of online discussions, when contrasted with less interested or knowledgeable participants (Catellani & Alberici, 2012; Catellani, Milesi, & Alberici, 2014; Kahn & Kenney, 1999; Milesi, 2016). Research into online deliberation, and on its effects on political engagement, could certainly help in this direction (see Halpern & Gibbs, 2013).

This study, to be certain, is in large part explorative. To corroborate our results, the investigation should be extended to other movements and contexts, and should involve larger samples. We also acknowledge that the current study considered only some of the specific features of online interactions. Further research could focus on other well-known characteristics of communicating through social media (e.g., Halpern & Gibbs, 2013), investigating their interaction with the psychosocial motives for collective action. For example, the perceived degree of civility of online discussions (i.e. the promoted respect and tolerance among users) could moderate the effect of action-relevant emotions (e.g. anger, contempt) on politicized identity and collective action. Moreover, it must be acknowledged that there are different degrees of both participation in online interactions and engagement in online discussions. The questions we used in the present research may not have captured the full range of these factors. Not only can people just read the discussions as they unfold without intervening; they also can participate in the interactions in a variety of ways (e.g. asking or giving information, asking or making suggestions), and with different levels of intensity (e.g. Vaccari et al., 2015). Future research should investigate how these different ways and levels of engagement in online discussions interact with psychosocial predictors of collective action in influencing politicized identity and collective action.

Despite these limitations, our findings suggest it is worthwhile continuing to investigate those features of contemporary CMC that may be relevant to shaping the meaning of people’s collective identities, thus sustaining or weakening their willingness to take part in collective actions.
